# Facing others’ misfortune: Personal distress mediates the association between maladaptive emotion regulation and social avoidance

**DOI:** 10.1371/journal.pone.0194248

**Published:** 2018-03-21

**Authors:** Delphine Grynberg, Belén López-Pérez

**Affiliations:** 1 Univ. Lille, UMR 9193, SCALab, Sciences Cognitives et Sciences Affectives, Lille, France; 2 Department of Psychology, Hope Park, Liverpool, Liverpool Hope University, Liverpool, United Kingdom; University of Bologna, ITALY

## Abstract

Previous research has linked the use of certain emotion regulation strategies to the vicarious experience of personal distress (PD) and empathic concern (EC). However, it has not yet been tested whether (1) vicarious PD is positively associated with maladaptive emotion regulation strategies, (2) vicarious EC is positively associated with adaptive emotion regulation strategies or whether (3) PD and EC mediate the link between emotion regulation and reports of approach/avoidance in response to a person in distress. To that end, we assessed people’s reports of PD (i.e., anxious, troubled and upset) and EC (i.e., concerned, sympathetic and soft-hearted) in response to a video depicting a person in a threatening situation (*n* = 78). Afterwards, we assessed participants’ reports of avoidance and approach with regard to the character and their disposition to use maladaptive and adaptive emotion regulation strategies. Results showed that both PD and EC were positively related to maladaptive strategies and negatively related to adaptive strategies, and that the association between maladaptive regulation strategies (i.e., rumination) and the willingness to avoid the person in distress was mediated by reports of greater PD. This study thus expands previous evidence on the relationship between maladaptive regulation strategies and affective empathy and provides novel insights into the main role that PD plays in the association between maladaptive strategies and social avoidance.

## Introduction

In the field of emotion regulation, most research has focused on its intrapersonal outcomes. As a result, the interpersonal domain has been neglected so far. The present research sought to fill this gap by examining the association between emotion regulation strategies, either adaptive or maladaptive, affective empathy and social behaviors.

### Emotion regulation and intrapersonal outcomes

Emotion regulation corresponds to a set of processes by which individuals assess and influence their own emotions, when they experience them, and how they express them [1]. According to the main theoretical model of emotion regulation (i.e., Gross’ Process Model of Emotion Regulation), strategies can be differentiated in terms of the moment they are implemented, either prior or after the full elicitation of the emotional response [1]. Besides this model, it has been proposed that emotion regulation strategies may also be classified as either more adaptive or maladaptive strategies [2–4]. Previous research has shown that emotion regulation strategies may have beneficial or detrimental effects on individuals’ functioning, in terms of affect, behavior and cognition, and their relationships to mental and physical health [1,5–7]. Putatively adaptive emotion regulation strategies such as cognitive reappraisal, acceptance and problem-solving have been associated with adaptive outcomes, including reduced experience of negative affect [8] and diminished cardiac reactivity [9]. On the other hand, putatively less adaptive emotion regulation strategies such as the suppression of the emotional experience or rumination have been associated with negative outcomes, including memory difficulties [10], increases in sympathetic activation [11], depression [2] and anxiety disorders [12].

### Emotion regulation and interpersonal outcomes

Even though these previous findings emphasize the main role of emotion regulation on intrapersonal outcomes, there is limited evidence in favor of interpersonal outcomes of emotion regulation. So far, most research has focused on the intrapersonal effect of relying on certain emotion regulation strategies. Little attention has been paid to how emotion regulation strategies modulate interpersonal functioning, despite the relevance of this research question in terms of the protective role of satisfactory social relationships. For instance, low empathic individuals report less satisfactory relationships [13], more loneliness [14] and less social support [15], which are known to deteriorate health and to increase the likelihood of mortality [16–18]. Research on the relation between emotion regulation and social functioning has shown that certain regulation strategies impact social support, social cognition and the quality of social interactions [19,20]. For instance, frequent use of reappraisal is associated with high peer-rating of likeability [19], whereas suppressing the expression of one’s own emotions during social interaction leads to higher physiological arousal in the partner [10]. Surprisingly, research examining the relationship between adaptive vs maladaptive emotion regulation strategies, empathy and social behaviors is sparse. The present research thus sought to better understand the interpersonal consequences of emotion regulation in terms of affective empathy and social behaviors.

### Emotion regulation, affective empathy and social behaviors

Before presenting the relevance of considering how emotion regulation is associated with affective empathy and social behaviors, these concepts should be defined. Empathy is a multidimensional construct that involves both affective and cognitive components [21,22]. The cognitive component is defined as the ability to take the perspective of others into account in order to understand and predict their mental states [23,24]. With regard to affective empathy, personal distress (PD) and empathic concern (EC) are generally considered as the two main possible vicarious emotional responses to others’ misfortune [25]. Whereas EC is defined as other-oriented and comprises feelings of warmth and sympathy, PD is defined as self-oriented and comprises feelings of discomfort and anxiety when facing another in need [25,26]. According to Batson [26], these two dimensions correspond to distinct latent factors which show either no correlation [27] or small-to-moderate correlations [28]. Measurement of these two vicarious emotional responses is based on either dispositional (e.g., Interpersonal Reactivity Index [21]) or situational affective responses to someone in distress. At a situational level, EC and PD are generally measured with emotion terms describing the current emotional experience of the participants. PD scores are derived from adjectives such as alarmed, grieved, upset, worried, disturbed or troubled whereas EC scores are derived from adjectives such as sympathetic, compassionate, moved or tender [26]. To better understand the role of emotion regulation in affective empathy, we focus on situational EC and PD. This enables the evaluation of transitory and actual measures of affective empathy, thus reducing the impact of self-representation or memory bias.

With regard to the associations between emotion regulation, EC and PD, Eisenberg and collaborators suggested that the way in which people regulate their own emotional experience may play a significant role in an individual’s vicarious emotional response (e.g. [29]).They found that greater abilities to control emotional responses are associated with reports of greater EC and of lower PD [30].These results are supported by more recent findings that individuals who generally experience EC tend to regulate more actively their emotional responses to pictures of people in pain, whereas those who generally experience PD do not tend to regulate actively their emotional responses [31]. Moreover, the relationship between emotion regulation and affective empathy has been recently supported by significant correlations between dispositional measures of regulation and PD (e.g. [32,33]).

Nonetheless, although these studies are a good first step in the study of emotion regulation and affective empathy, they either rely on dispositional measures of EC and PD rather than on situational contextualized emotional responses or they used an index of emotion regulation that comprises several regulation strategies considered as adaptive (i.e., attention shift, distracting) and maladaptive (i.e., emotional suppression). To overcome these limitations, a recent study showed that participants under rumination instructions experienced higher levels of PD in response to someone’s distress than participants using a more adaptive strategy (i.e., reappraisal) who experienced greater EC [34]. The authors manipulates emotion regulation strategies with experimental instructions and a priming procedure in response to a picture depicting a sick child in a hospital bed with a facial expression of pain. They showed that participants reported higher EC in the reappraisal condition compared to the rumination condition, whereas they reported higher PD in the rumination condition compared to the reappraisal condition.

Nevertheless, these studies have overlooked the link between other forms of maladaptive and adaptive emotion regulation and PD and EC, thus precluding any other potential links between these vicarious emotions and other regulation strategies. For instance, a recent meta-analysis showed that accepting the emotion or taking a detached perspective from the stimulus have positive effect on emotional responses [35].

Furthermore, to our knowledge, no previous research has explored whether affective empathy accounts for the association between emotion regulation and the behavioral correlates of empathy, i.e. approaching/avoiding the person in need. Several studies have shown that the tendency to feel compassion motivates us to improve the well-being of others in an altruistic way, i.e., aiming to help others diminish their distress independently of the advantages we can gain from the situation, and is associated with fewer antisocial behaviors, whereas the tendency to feel distressed reduces supporting behaviors [36–38].

These behavioral correlates are essential in human relationships as prosocial behaviors (e.g., volunteering) allow for social cohesion [39] and are associated with better personal health outcomes [40], whereas avoidance behaviors may have a detrimental social impact. For instance, research suggests that socially avoidant women (i.e., avoiding gaze) are perceived as less agreeable and conscientious than those who have a direct gaze [41]. Similarly, selfish behavior (e.g. unfair behavior in a monetary game) has been shown to reduce empathic responses from other players [42], supporting the main role of approach/avoidance behavior to promote social relationships.

In summary, emotion regulation has been examined so far mainly through its intrapersonal outcomes, whereas its impact on interpersonal dimensions such as affective empathy and social behaviors has received little attention. As previously suggested, because adaptive emotion regulation strategies have positive intrapersonal outcomes (e.g., mental and physical health) and negative strategies are associated with poor mental and physical outcomes (e.g., [5–7]), we sought to better understand whether they also influence core interpersonal functions, namely affective empathy (EC and PD) and social behaviors (approach and avoidance).

### The present research

The main aim of the study was to provide a deeper understanding of the role of dispositional adaptive and maladaptive regulation strategies (1) in the experience of PD and EC when facing someone in distress and (2) in the willingness to avoid or approach this person. To determine the deleterious and beneficial emotion regulation strategies for interpersonal functioning, we tested various adaptive and maladaptive strategies. The secondary aim was to test whether affective empathy mediates the association between emotion regulation and avoidance/approach. We hypothesized that maladaptive regulation strategies would be positively associated with PD, and that adaptive strategies would be positively associated with EC (e.g., [31,34]). Furthermore, because EC is associated with altruistic motivation and helping behaviors [25], we expected a positive correlation between EC and approach behaviors. On the other hand, because PD is associated with egoistic motivation and less helping behaviors (e.g., if escaping is easy [25]), we expected a positive correlation between PD and avoidance. Finally, we expected that maladaptive regulation strategies would be related to higher avoidance/lower approach, through reports of greater PD. This hypothesis emerged from findings showing that frequent use of maladaptive regulation strategies (i.e., suppression) is associated with reports of lower prosocial tendency [43]. However, because previous findings revealed no association between greater prosocial tendency neither with reappraisal training nor with frequent use of reappraisal [43,44], we did not expect any adaptive regulation strategies to be related to lower avoidance/higher approach, through reports greater of EC.

## Method

### Participants

In this study 81 participants (57 females) aged between 18 and 67 years (*M* = 25.68; *SD* = 10.88) participated in exchange of a credit or a monetary reward of £4. Participants were university students and members of the public recruited through the paid participation pool systems at one of the authors’ institution. Inclusion criterion was to be above 18 years old. The number of participants was determined based on expected medium correlations (*r* = .30) at a significance level of α = .05 and a power of 1-β = .085. Three participants were removed from the analyses because they were outliers (+3SD) in terms of age. This was the only exclusion criterion. The statistical analyses were thus performed among the remaining 78 participants (55 females) aged between 18 and 57 years (*M* = 24.28; *SD* = 8.32).

### Material

#### Video

Participants watched a 2-minute video clip taken from Barraza and Zak [45]. The video shows a father describing his experience with his 2-year-old son who suffers from a terminal brain cancer. This video was chosen because of its effect on affective responses and oxytocin production [45].

#### Situational personal distress

(based on [26]) required from participants to indicate on a scale ranging from 1 (strongly disagree) to 7 (strongly agree) whether they felt alarmed, troubled and upset (PD; α = .80) and concerned, sympathetic and soft-hearted (EC; α = .64) while watching the video.

#### Avoidance response 3-item questionnaire

[46] required participants to indicate on a scale ranging from 1 (strongly disagree) to 7 (strongly agree) to what extent (1) they “wanted to be completely unassociated with the child”, (2) they “*wanted to disappear from the situation*”, and (3) they “*did not want to be associated in any way with the child*”.

#### Approach response

participants were asked whether they wished to receive more information about Ben (the child). If their response was affirmative, they had to indicate their email address to receive further updates. This was considered as an objective measure of approach.

#### Short cognitive emotion regulation questionnaire

(CERQ-short [3]) is an 18-item scale designed to evaluate the conscious cognitive aspects of emotion regulation. Participants were instructed to evaluate on a Likert scale (from 1 = almost never to 5 = almost always) the frequency they use each regulation strategy. Nine emotion regulation strategies are measured and can be grouped into adaptive (acceptance, positive refocusing, planning, reappraisal, and putting into perspective) and maladaptive (self-blame, other-blame, rumination, and catastrophizing) strategies. *Acceptance* refers to thoughts of resigning oneself to what has happened; *Positive Refocusing* assesses thinking about positive experiences instead of thinking about the actual event; *Planning* evaluates thinking about what steps to take and how to handle the negative event; *Reappraisal* measures thoughts of giving the event a positive meaning in terms of personal growth and *Putting into Perspective* refers to downgrading the importance of the event. Regarding maladaptive strategies, *Self-blame* evaluates thoughts of putting the blame for what you have experienced on yourself; *Other-blame* assesses thoughts of putting the blame of what one has experienced on the environment or on another person; *Rumination* refers to thinking about the feelings and thoughts associated with the negative event and *Catastrophizing* measures thoughts of explicitly emphasizing the terror of what one has experienced. We also calculated an index of adaptive and maladaptive emotion regulation strategies averaging the corresponding scales. The Cronbach alphas were respectively α = .91 and α = .71 in the present sample.

### Ethical statement

Ethical approval: All procedures performed in studies involving human participants were in accordance with the ethical standards of Plymouth University Research Ethics Committee, permit number FREC-PSY456-15 and with the 1964 Helsinki Declaration and its later amendments or comparable ethical standards. Informed consent was obtained from all individual participants (i.e., written document mentioning their right to withdraw from the study at any time and that their data would remain anonymous).

### Procedure

Participants were tested individually. Once they had signed the consent form, they were informed they would watch a video about a random topic and then would be asked to answer some questions about it. All participants then watched the 2-minute video clip and afterwards completed the situational personal distress scale, the three-item scale to assess self-report avoidance and the approach question. Finally, they completed the CERQ. At the end, they were fully debriefed about the study. The whole study was computer-based and lasted 30 minutes.

### Data analysis

Statistical analyses were performed using the SPSS software package. Skewness and kurtosis values were below 2 for all variables, suggesting that they were normally distributed. There were outliers as Z scores in each variable were below +/- 3 SD. The association between all variables was investigated with Pearson correlations, except for the measure of approach (i.e., dichotomous variable), for which we used Kendall’s tau-b. We corrected for multiple comparisons by using the Benjamini–Hochberg procedure to hold the false discovery rate at 5% for the 69 correlations. We thus considered only correlations that were significant at *p*<.017. We also tested whether participants used some emotion regulation strategies more frequently than others (9 variables) and whether participants reported more or less personal distress than empathic concern (2 variables) with a Repeated Measures ANOVA with regulation strategies and vicarious emotional responses as within-subject factors. Finally, we examined whether affective empathy mediated the associations between emotion regulation and the willingness to avoid or approach the person in distress by running Hierarchical Linear Regressions. We entered affective empathy on the first step of the regression analysis and emotion regulation strategy on the second step. Willingness to avoid or approach was the outcome variable.

## Results

### Descriptive data

Means and standard deviation of all variables are presented in Table 1 (data available, S1 File). A repeated measures ANOVA with regulation strategies as within-subject factor showed a main effect of regulation strategies (*F*(8, 616) = 48.21; *p* <.001; *Partial eta²* = 0.39), suggesting that participants used strategies to a different extent. Contrast analyses revealed that whereas reappraisal was the most frequently used strategy, blaming others was the least used. All other comparisons between strategies are mentioned in Table 1 (i.e., superscripts next to the means). Regarding affective empathy, a repeated measures ANOVA showed that participants reported more EC than PD in response to the video (*F*(1, 77) = 42.74; *p* <.001; *Partial eta²* = 0.36).

**Table 1:** Correlations and descriptive statistics (mean and SD) of affective empathy, avoidance, approach and emotion regulation strategies.

					Correlations					
		Mean	SD	Range	Personal Distress	Empathic Concern	Avoidance Question 1	Avoidance Question 2	Avoidance Question 3	Approach (Kendall’s tau-b)
Personal Distress		4.73	1.25	2.67–7.00	-	.45***	.09	.34**	.03	.17
Empathic Concern		5.61	0.99	2.67–7.00	-	-	-.03	.11	.00	.16
Avoidance	Question 1	2.88	1.86	1.00–7.00	-	-	-	.70***	.93***	-.40***
	Question 2	2.71	1.52	1.00–7.00	-	-	-	-	.71***	-.33**
	Question 3	2.81	1.90	1.00–7.00	-	-	-	-	-	-.45***
Approach		43,6%								
CERQ-short	Self-blame	2.34^e^	0.69	1.50–4.00	.23	.13	.05	.02	.05	-.31**
	Other-blame	2.10^f^	0.68	1.00-.4.50	.51***	.43***	.23	.32**	.17	.14
	Rumination	3.48^c^	0.38	3.00–4.50	.46***	.36**	.13	.27*	.10	-.09
	Catastrophizing	2.77^de^	1.01	1.00–4.00	.33**	.44***	.11	.19	.08	.29**
	Acceptance	3.82^b^	0.75	2.50–5.00	-.56***	-.49***	.05	-.03	.08	-.11
	Positive Refocusing	2.93^d^	1.07	1.00–5.00	-.52***	-.61***	.02	-.12	.03	-.10
	Planning	3.9^ab^	0.80	2.50–5.00	-.27*	-.32**	-.01	-.15	.00	.00
	Reappraisal	4.03^a^	0.76	2.50–5.00	-.30**	-.25	.04	-.02	.04	-.04
	Putting into perspective	3.50^c^	1.12	2.00–5.00	-.52***	-.11	.12	-.02	.17	.04

* *p*<.017

** *p*<.01

*** *p*<.001

^*abcdef*^ Superscripts indicating significant difference between means of different strategies at a significant level of *p*<.05

### Affective empathy, emotion regulation and avoidance/approach

As shown in Table 1, PD and EC were positively associated with maladaptive regulation strategies and negatively with adaptive strategies. Moreover, there were positive correlations between other-blame, rumination and participants’ self-reported avoidance (i.e., Question 2, the desire to disappear from the situation). Moreover, self-blame was negatively associated with approach. Finally, PD was positively correlated with participants’ self-reported avoidance (i.e., Question 2). All other correlations between affective empathy, on the one hand, and emotion regulation and social avoidance/approach, on the other, were not significant.

### Mediation analyses (Fig 1)

Mediation analyses were performed to examine whether PD mediated the associations between rumination/other-blame and self-report avoidance measured by Question 2. Regression analyses showed that after adding personal distress as a mediator, neither rumination nor other-blame predicted avoidance any more (rumination, β = .14, B = .56, SEB = .48 *t*(77) = 1.17, *p* = .25; F(2, 77) = 5.73; p = .005; other-blame, β = .20, B = .37, SEB = .23, *t*(77) = 1.59, *p* = .12; F(2, 77) = 6.39; p = .003). Importantly, the association between PD and avoidance remained significant only when controlling for rumination (β = .28, B = .34, SEB = .15, *t*(77) = 2.27, *p* = .026). When controlling for other-blame, PD was not significantly associated with avoidance (β = .24, B = .29, SEB = .42, *t*(77) = 1.95, *p* = .06), suggesting that personal distress mediated the association between CERQ rumination and avoidance. The reverse mediation model with PD as the dependent variable, avoidance as the mediator, and CERQ-rumination as the independent variable was not significant, as rumination still predicted PD after controlling for avoidance (β = .40, *B* = 1.30, *SEB* = .33 *t*(77) = 3.88, *p*<.001; *F*(2, 77) = 13.87; *p*<.001). Owing to the wide age range, we conducted additional analyses controlling for age. Results showed no significant impact on p-values.

**Fig 1:**
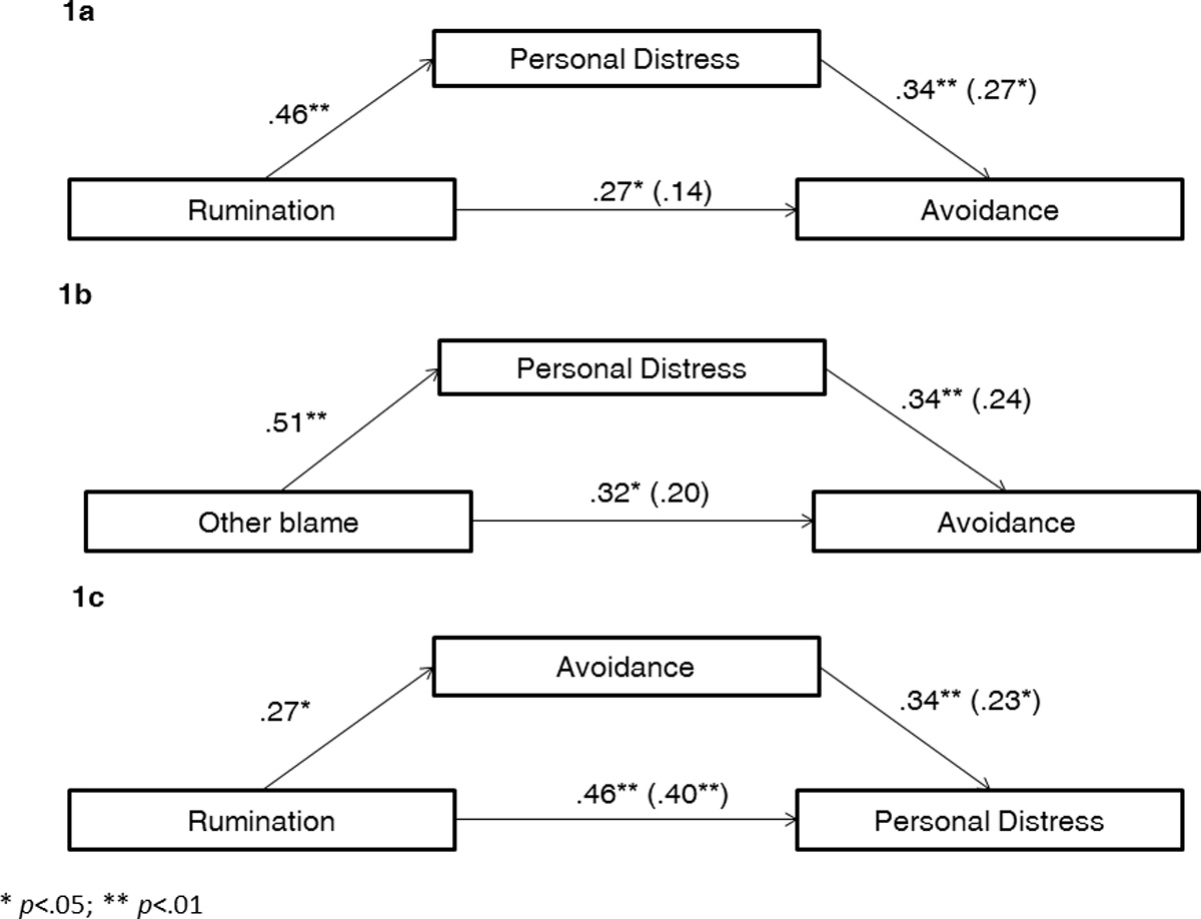
Mediational models: The effect of CERQ rumination (Fig 1A) and CERQ other-blame (Fig 1B) on avoidance (*“Desire to disappear from the situation”*) through personal distress and the effect of CERQ rumination on personal distress through avoidance (*“Desire to disappear from the situation”*) (Fig 1C).

## Discussion

The present study examined the links between adaptive and maladaptive regulation strategies, affective empathy and avoidance/approach tendencies. Specifically, we aimed to examine whether frequent use of maladaptive emotion regulation strategies was associated with avoidance behaviors through report of greater personal distress. To this end, participants were instructed to rate their feelings (i.e., personal distress and empathic concern) in response to a person in distress. Afterwards, they had to evaluate their willingness to avoid or approach the distressful situation. Emotion regulation strategies were assessed by a self-report questionnaire that examine the frequency at which individuals used various adaptive and maladaptive strategies.

### Emotion regulation and affective empathy

Results revealed that participants often used acceptance, planning and reappraisal to regulate their emotions. On the other hand, they relied only sometimes on blaming others to regulate their own emotions. This is in line with previous findings showing that individuals rely more on adaptive than on maladaptive strategies [3]. It also suggests that emotion regulation strategies may be divided into either more adaptive or maladaptive strategies in terms of their beneficial or detrimental impact on mental and physical health [1–3,5–7]. Putatively adaptive emotion regulation strategies such as cognitive reappraisal, acceptance and problem-solving have been associated with adaptive outcomes, including reduced experience of negative affect [8] and diminished cardiac reactivity [9].

Regarding affective empathy, participants reported more EC than PD in response to the video, suggesting that the video was not too overwhelming for them. Concerning the links between emotion regulation and affective empathy, the results support our hypothesis that PD is positively related to the frequent use of maladaptive emotion regulation strategies and negatively to adaptive emotion regulation strategies. We show that participants who reported greater PD in response to the person depicted in the video also reported frequent use of rumination, other-blame, catastrophizing and less frequent use of acceptance, positive refocusing, planning, reappraisal and putting things into perspective. The positive association between PD and rumination supports previous findings showing that the tendency to ruminate (measured by the Rumination-Reflection Questionnaire, e.g., “*I tend to ruminate or dwell over things that happen to me for a really long time afterward”*) was associated with report of greater personal distress (based on the IRI, [21]) [47]. The present findings are also in line with López-Pérez and Ambrona’s findings that the induction of rumination thoughts (i.e., *“think repetitively about the experienced feelings and thoughts related to those feelings*, *by focusing the attention on one’s own emotions*”) leads to greater report of PD than EC [34]. These findings and the present ones suggest that focusing on the broad experience of a negative emotion, its causes and consequences may also intensify one’s own negative mood [2]. It has indeed been shown that rumination prospectively predicts symptoms and diagnoses of anxiety and depression [2], supporting the deleterious effect of rumination on negative affect. Therefore, one can assume that participants who tend to ruminate may experience more overwhelming negative feelings, irrespective of their social dimension.

Furthermore, PD was linked to all other maladaptive regulation strategies, namely catastrophizing, self-blame and other-blame. This is in line with previous research on the role of catastrophizing thoughts in PD feelings in response to others’ pain [48]. Regarding self-blame, the result is coherent with previous research linking self-criticism, i.e. people’s tendency to make negative self-evaluative comments, to PD [26]. Finally, concerning the association between other-blame and PD, this may be related to previous research linking PD and a belief in a just world that leads to a lack of helping (i.e., a cognitive bias which consists in blaming people for their own problems, regardless of the situation) [49].

Whereas the present research supports multiple findings about PD and maladaptive strategies, it is to our knowledge the first study to show that PD is negatively associated to various adaptive strategies of reappraisal, acceptance, positive refocusing, and putting things into perspective. Specifically, although the design was correlational, our findings extend those of López-Pérez and Ambrona [34] by showing that the frequent use of other adaptive regulation strategies might reduce PD. In other words, being able to accept the situation as it is (*acceptance*), to think about it differently either by focusing on positive aspects (i.e., *reappraisal*) or by downgrading its importance (i.e., *putting things into perspective*) or not focusing on the situation itself (*positive refocusing*, thinking about other positive experiences; *Planning*, thinking about how to handle the negative event) is associated with lower distress in response to others’ misfortune. Therefore, more adaptive (maladaptive) regulation strategies may have beneficial (detrimental) effects. A body of evidence demonstrates that at an intrapersonal level, maladaptive strategies are positively associated with depression, anxiety and with greater distress responses to unpleasant situations (e.g., [50–52]). The present study thus extends these findings by showing their significant associations with interpersonal factors. Further studies should thus compare the affective responses at both intra- and interpersonal levels to provide an in-depth understanding of the specificity of empathic responses.

Surprisingly and in contradiction with our hypotheses, EC showed exactly the same pattern of results as those found between PD and emotion regulation. The present findings are thus in contradiction with previous data showing that under instructions of reappraisal, individuals report greater EC than under instructions of rumination [34] and that disposition EC is associated with disposition regulation control [53]. Various arguments can be made to explain the present pattern of results. First, measuring situational EC as a core specificity of affective empathy (e.g., sympathy and warm feelings as well as concern for others) may be more difficult that targeting situational PD (as indicated by its low internal consistency). For instance, Lamm and colleagues [54] showed that reappraisal influences the subjective report of PD but not EC. In their study, participants were instructed to observe facial expressions of pain. They were all told that the pain inflicted on the person in the video was part of a medical protocol. Half of the sample was informed that the treatment was effective, while the other half was told that it was not. The effect of reappraisal was thus measured by manipulating the effectiveness of the treatment. Participants in the "non-effective" group reported greater distress than those in the "effective” group. However, there was no effect of reappraisal on the subjective reports of empathic concern. Other factors may also account for the counterintuitive association between EC and emotion regulation. For instance, the video may have induced intense emotions, which led participants to reporting strong emotional responses in general. Finally, EC and PD may measure a common latent factor such as emotional reactivity, as indicated by the moderate correlation between EC and PD, and may thus share more features that theoretically argued and empirically demonstrated (e.g.,[45]). It is finally worth mentioning that not all studies found a relationship between disposition EC and any measure of emotion regulation [32,55] and that some studies even found a negative association between situational EC and emotion regulation [55].These elements (i.e., arousing video, common latent factor, and weak EC internal consistency) may also account for the absence of correlations between EC and both avoidance and approach behaviors. Another explanation may lie in the content of the first and the third avoidance questions. These two questions indeed referred to the child, while the character depicted in the video was the father talking about his son. Therefore, EC and PD were most probably experienced in response to the father (and not the child), accounting for the non-significant associations between EC, PD and questions 1 and 3.

Finally, avoidance and approach behaviors were not predicted by any adaptive regulation strategies. Specifically, the absence of significant correlations between these behaviors and reappraisal or putting into perspective were surprising, considering the positive effect of these strategies on emotional responses [35]. To our knowledge, few studies have examined the links between emotion regulation strategies and prosocial behaviors and the findings are not consensual. For instance, a study showed that reappraisal is not associated with prosocial behaviors, whereas it moderates the extent to which these behaviors are predicted by affective empathy [43]. Among children and teenagers, some data indicate that higher regulation strategies are associated with self-reported prosocial behavior but not with teachers’ reports of prosocial behaviors [56]. Based on parents’ reports, there are significant associations between emotion regulation abilities and prosocial behaviors [57]. Finally, a study suggested that negative affect induction moderates the effect of emotion regulation on prosocial behaviors [58]. Therefore, further studies are needed to understand whether the effect of emotion regulation strategies on prosocial behaviors is significant only for some strategies or whether they act rather as a moderator.

In sum, further studies should better apprehend empathic concern as a distinct dimension of affective empathy and determine more adequate ways to differentiate EC and PD based on subjective self-reports, physiological indices such as sympathetic (skin conductance) and parasympathetic activity (vagal activity) or facial expressions.

### Emotion regulation, affective empathy and social avoidance/approach

With respect to avoidance, results showed that the maladaptive strategy of blaming others was associated with the tendency to avoid the situation in which a person is in distress. Holding people responsible for what they experience may strengthen individuals’ willingness to distance oneself from others’ problems at both affective and behavioral levels. On the other hand, it is possible that people who distance themselves from other people (i.e., leading them to avoid a person in distress) naturally hold other people more accountable for their actions. Finally, another hypothesis is that believing in a just world may cause people to distance themselves from others and hold them accountable for what they experience. In this regard, previous literature has shown less avoidance (i.e., more helping) when victims were described as not responsible from their own problems (e.g.,[49]).

Regarding rumination, the present study shows that individuals who frequently use rumination as a strategy to regulate their emotion report greater willingness to avoid the person in distress, owing to greater reports of PD. Recent studies support the association between rumination and avoidance. For instance, higher levels of grief-related rumination are associated with a strong implicit loss avoidance (i.e., pushing a joystick away from oneself in response to a picture of the deceased relative presented together with a loss-related word) and to less overall time spent looking at this picture-word combination [59,60]. Moreover, rumination has been associated with reports of frequent behavioral avoidance [61], suggesting that rumination is an important predictor of social avoidance. Importantly, we show for the first time that situational PD may account for this effect. To our knowledge, only two studies have investigated the role of empathy in the effect of emotion regulation on either prosocial behavior [56] or hostility [32]. However, they had shortcomings such as the use of dispositional measures and a global score of empathy and/or difficulties in emotion regulation. The present study is thus the first to suggest that participants who are frequently preoccupied by their feelings and thoughts associated with a negative event might have focused on their responses to the distressed person depicted in the video, which may have afterwards led them to experience greater distress. This distress may have consequently increased their willingness to avoid the situation in order to cope with it.

### Limitations

The study has some limitations regarding the cross-sectional design and the inference of causality. It did not allow us to examine whether the causality between emotion regulation and affective empathy is unidirectional. This is particularly important as it has been shown that while emotion regulation strategies may modulate vicarious emotional responses [34], they may also be modulated by them [62]. Although participants were not instructed to use specific regulation strategies while watching the video, our findings suggest that frequent use of maladaptive strategies may have harmful interpersonal effects. Based on previous results which support the links between dispositional and situational measures of catastrophizing [63] or emotional competences [64], one can hypothesize that dispositional measures of regulation may predict the situational use of these strategies. Further studies should also test more participants (and more male individuals) and use objective measures of avoidance and approach, which were mainly limited to self-report in the present study.

## Conclusion

In conclusion, we show that maladaptive emotion regulation strategies not only have an impact on PD but also on avoidance behavior when facing a person in need. Therefore, this study provides new research avenues that will allow the mechanisms that account for one’s own ability to cope efficiently with others’ suffering to be examined. It also suggests that by understanding better the link between emotion regulation, affective empathy and possible responses to others’ distress, we might be able to prevent responses such as “compassion burnout”, which is quite likely to occur in professionals dealing with others’ suffering on a daily basis such as nurses.

## Supporting information

S1 File Data. (XLSX)
